# Supplementary material for: Mechanism of collagen folding propagation studied by Molecular Dynamics simulations
Source: PLoS Comput Biol. 2021 Jun 8;17(6):e1009079. doi: 10.1371/journal.pcbi.1009079 (PMC8224937; doi:10.1371/journal.pcbi.1009079)
Supplement: S1 Fig — (PDF) [file pcbi.1009079.s001.pdf]

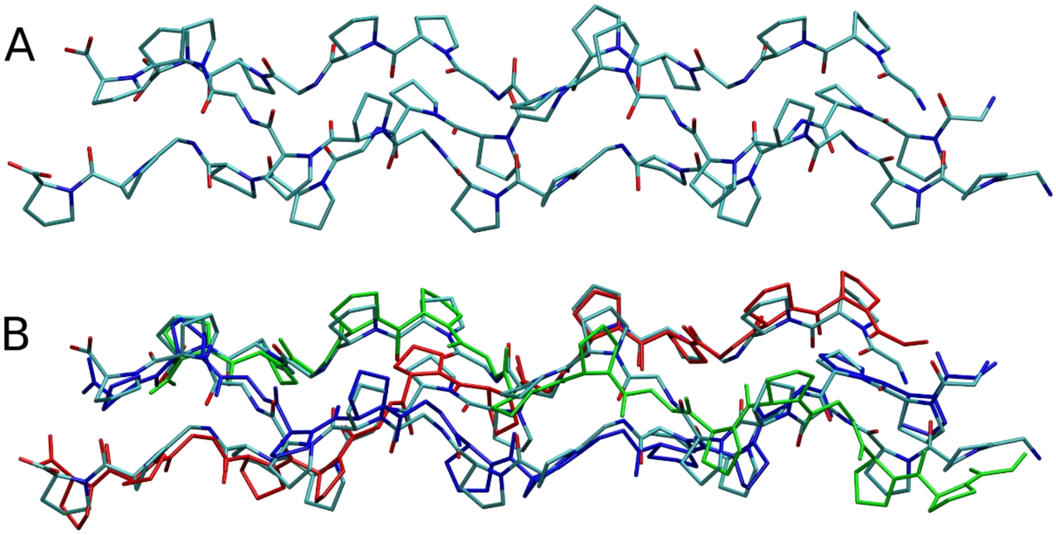

S1_Fig. (A) Structure of a folded triple helix peptide of 3 x (Gly-Pro-Pro)$_5$ sequence. (B) Superposition of crystal structure (light blue) and final peptide (each chain in different color) after folding simulation.
