# Supplementary material for: Mechanism of collagen folding propagation studied by Molecular Dynamics simulations
Source: PLoS Comput Biol. 2021 Jun 8;17(6):e1009079. doi: 10.1371/journal.pcbi.1009079 (PMC8224937; doi:10.1371/journal.pcbi.1009079)
Supplement: S2 Fig — (PDF) [file pcbi.1009079.s002.pdf]

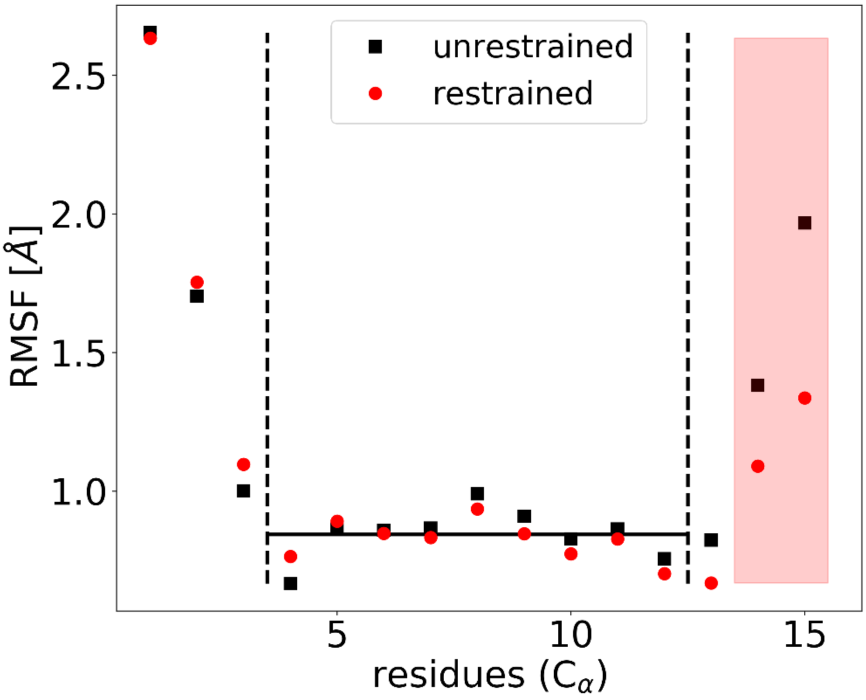

S2_Fig. Root Mean Square Fluctuations (RMSF) of an unrestrained and a restrained triple helix: The black squares represent the RMSF of the C_α atoms of the residues along the helix (mean of three residues, one of each strand). The N-terminus (left) and the C-terminus (right) show increased flexibility. The red circles represent the RMSF of a triple helix which was restrained on the two terminal residues at the C-terminus (red area). Clearly visible is that the restrained C-terminus is less flexible than the unrestrained terminus, but still more flexible than the middle part of the helix (between the dashed lines).
