# Supplementary material for: Mechanism of collagen folding propagation studied by Molecular Dynamics simulations
Source: PLoS Comput Biol. 2021 Jun 8;17(6):e1009079. doi: 10.1371/journal.pcbi.1009079 (PMC8224937; doi:10.1371/journal.pcbi.1009079)
Supplement: S3 Fig — (PDF) [file pcbi.1009079.s003.pdf]

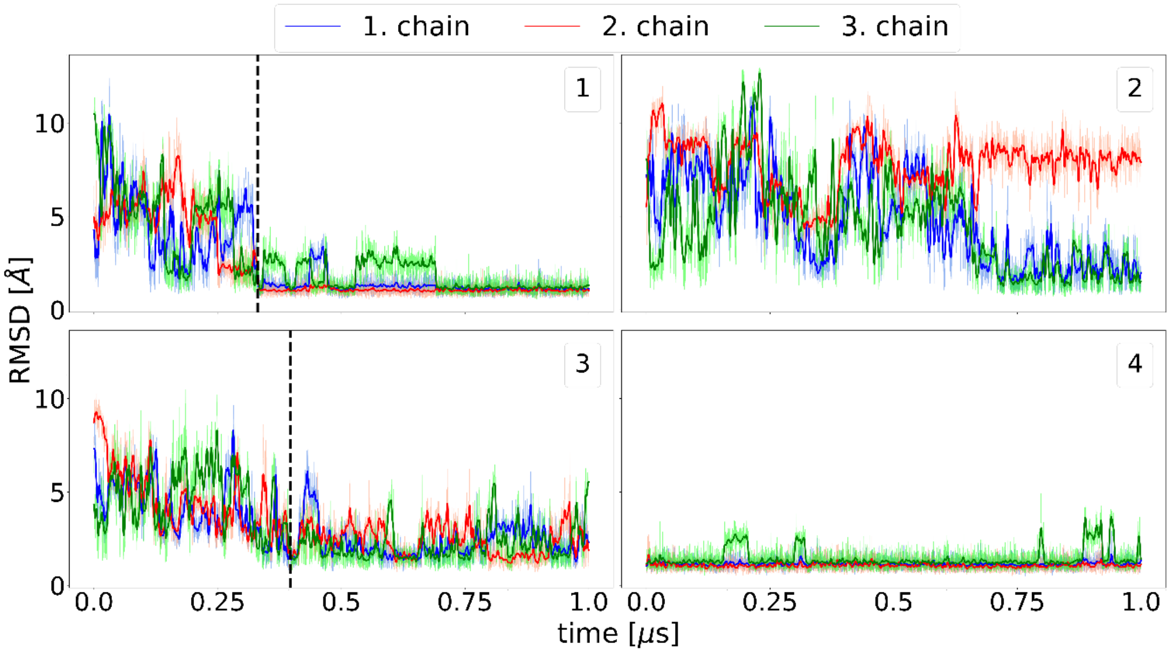

S3_Fig. RMSD of individual chains (with respect to the native collagen structure) vs. simulation time for 3 folding simulations (1-3) and one simulation starting from foled triple helix (4). The RMSD of each strand is visualized separately (blue, red, green). The thicker line shows the smoothed data (Gaussian filter, σ=25). The vertical dashed line marks the time point of (first) folding (RMSD of all chains below 2 Å). Example 1 indicates a successful folding of the wild type sequence. One chain in example 2 formed a loop in the second (red) strand in final part of the simulation and the RMSD of the chain remains at a high level. Example 3 shows a mutant with the 3 central Gly residues replaced by Ala. Although the RMSD temporarily reaches low values, no stable folding is observed. Finally, the 4th example shows the simulation starting from the native folded triple helix.
