# Supplementary material for: Mechanism of collagen folding propagation studied by Molecular Dynamics simulations
Source: PLoS Comput Biol. 2021 Jun 8;17(6):e1009079. doi: 10.1371/journal.pcbi.1009079 (PMC8224937; doi:10.1371/journal.pcbi.1009079)
Supplement: S4 Fig — (PDF) [file pcbi.1009079.s004.pdf]

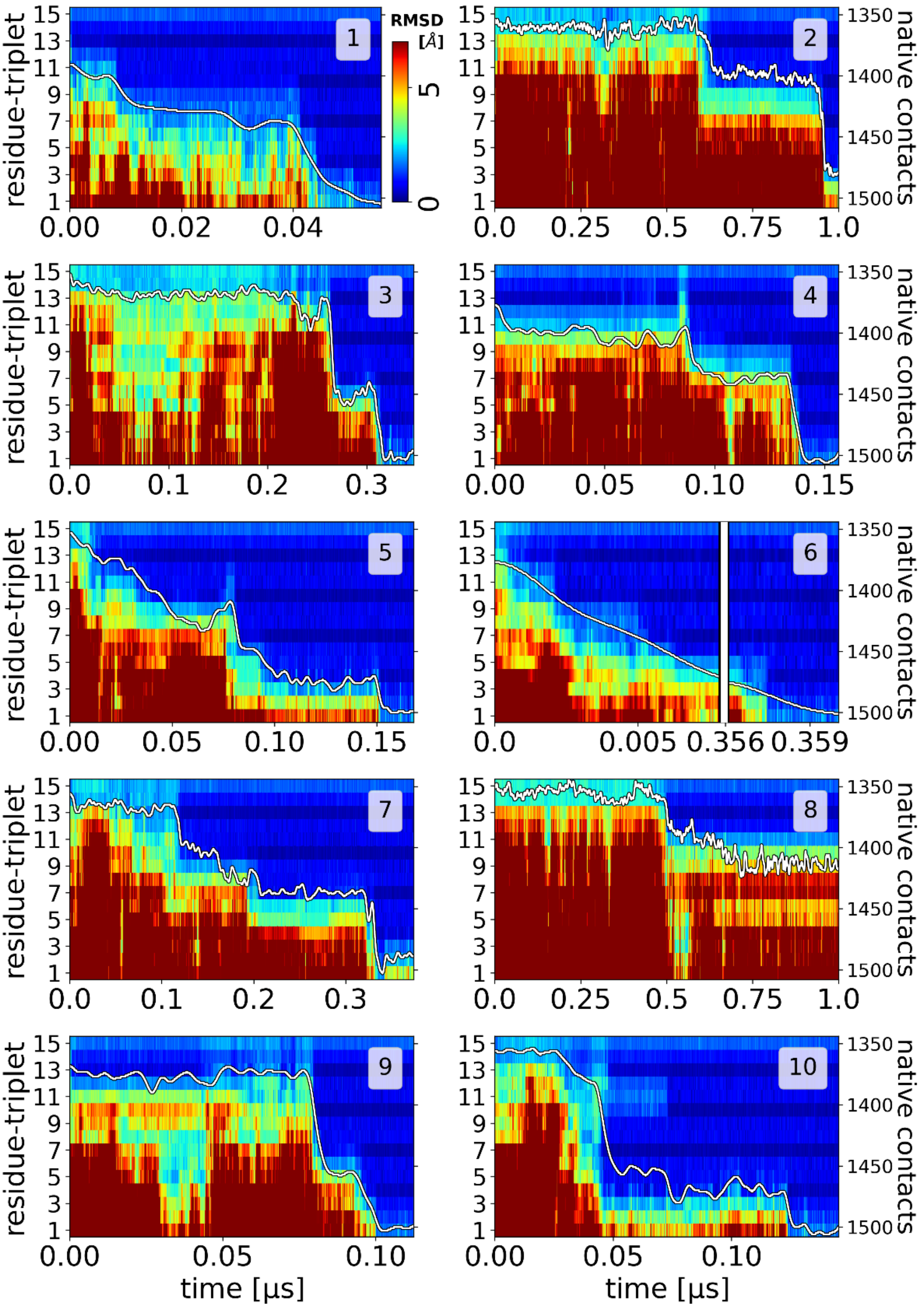


**S4_Fig.** RMSD of residue triplets of all WT simulations (see Fig. 1 in manuscript). Each simulation starts from an unfolded collagen peptide with an already formed nucleus at the C-terminus (top). Each labelled stripe (1-15) in the central plot represents a residue triplet (one residue with the same number of each strand, e.g. 3 Pro with the same number that are spatially close in the native state) along the three strands. The RMSD of each residue triplet relative to the native folded structure is indicated by a color-code (color bar in the first plot). A blue color represents sampled states close to native, whereas red color corresponds to an unfolded triplet structure. The y-axis on the right side of the plot represents the number of native contacts between the three strands (white line in the plot). All simulation ran for 1µs. To present the folding process in detail the time range of each graph was adjusted.
