# Supplementary material for: Mechanism of collagen folding propagation studied by Molecular Dynamics simulations
Source: PLoS Comput Biol. 2021 Jun 8;17(6):e1009079. doi: 10.1371/journal.pcbi.1009079 (PMC8224937; doi:10.1371/journal.pcbi.1009079)
Supplement: S5 Fig — (PDF) [file pcbi.1009079.s005.pdf]

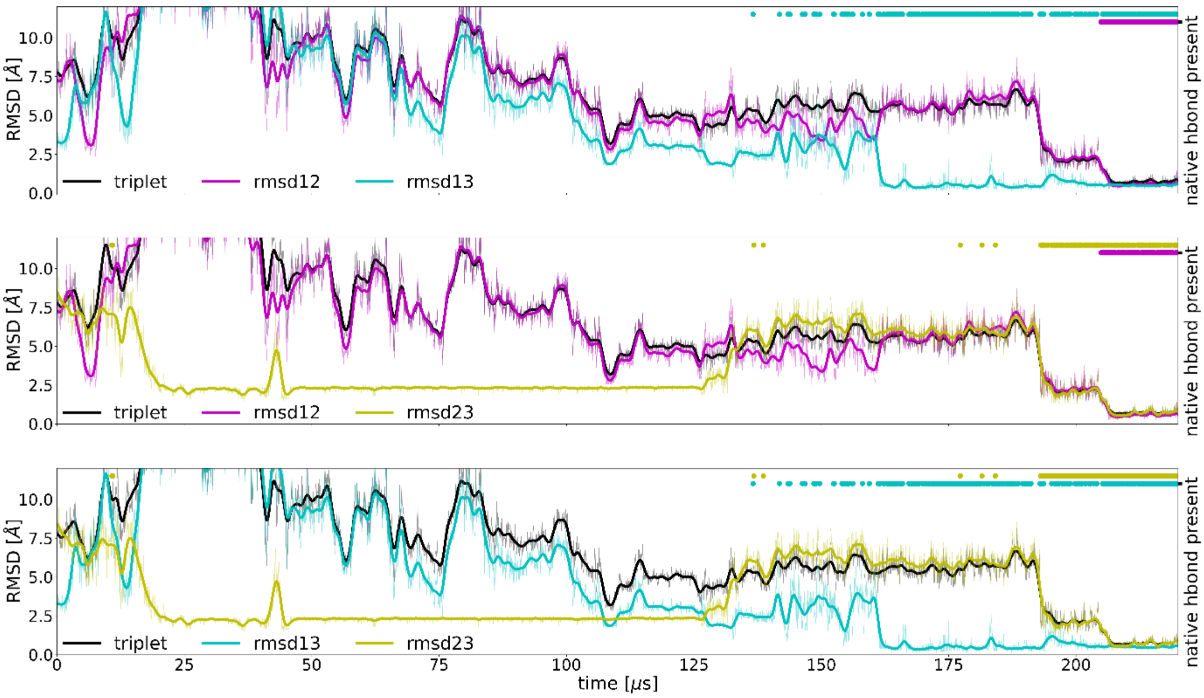

S5_Fig. Detailed example of a folding process at one point along the triple helix (residue 7 of chain A=1, B=2, C=3). Each plot visualizes the situation of one strand of the helix. The black curve shows the RMSD of all three strands. The colored curves indicate the pairwise RMSD of 2 selected chains. (magenta: 1&2, cyan: 1&3, yellow: 2&3). On the top of each plot the presence of the corresponding hydrogen bond is indicated by dots in the same colors. In the first half the 2nd and 3rd strand align to a metastable conformation, but the first strand does not join them. Also the corresponding hydrogen bond is not formed. After 125ns both strands separate again. In the last third strand 1 and 3 align to each other and the hydrogen bond is formed. Around 200ns the 3rd strand follows and the final and stable conformation is formed.
