# Supplementary material for: Mechanism of collagen folding propagation studied by Molecular Dynamics simulations
Source: PLoS Comput Biol. 2021 Jun 8;17(6):e1009079. doi: 10.1371/journal.pcbi.1009079 (PMC8224937; doi:10.1371/journal.pcbi.1009079)
Supplement: S6 Fig — (PDF) [file pcbi.1009079.s006.pdf]

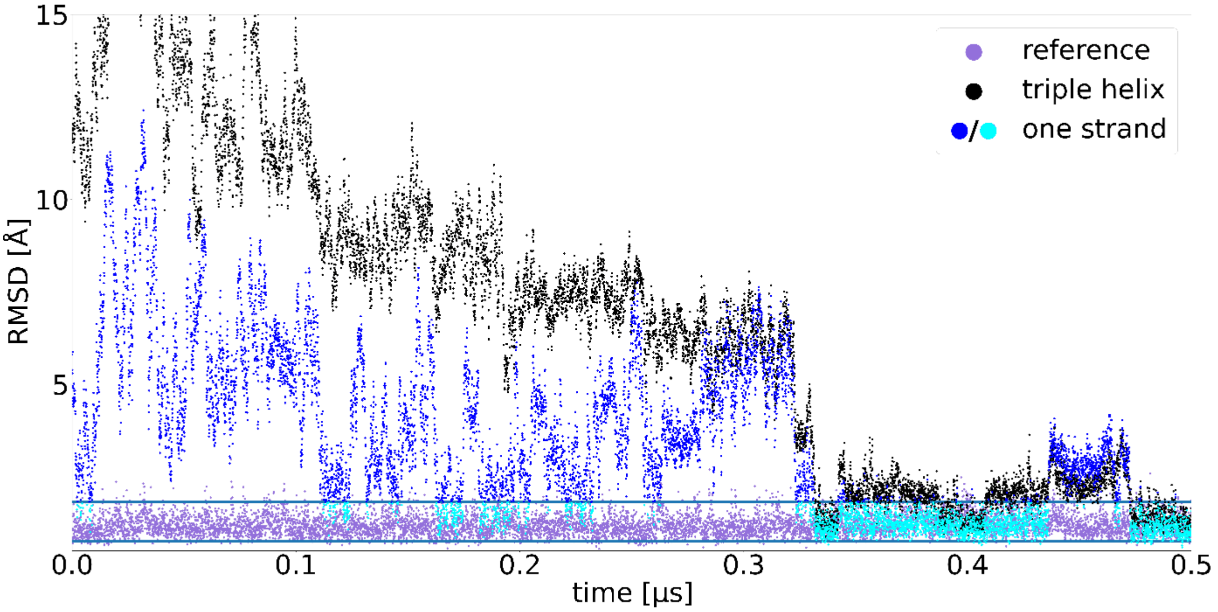

S6_Fig. Root mean square deviation (RMSD) of individual chains during triple helix folding. For an example folding simulation the RMSD (non-hydrogen atoms) with respect to the native triple helical conformation vs. time is indicated (black curve: RMSD vs time of complete structure; violet: simulation starting from the triple helix; dark/light blue: RMSD of separate chains.
