# Supplementary material for: Mechanism of collagen folding propagation studied by Molecular Dynamics simulations
Source: PLoS Comput Biol. 2021 Jun 8;17(6):e1009079. doi: 10.1371/journal.pcbi.1009079 (PMC8224937; doi:10.1371/journal.pcbi.1009079)
Supplement: S7 Fig — (PDF) [file pcbi.1009079.s007.pdf]

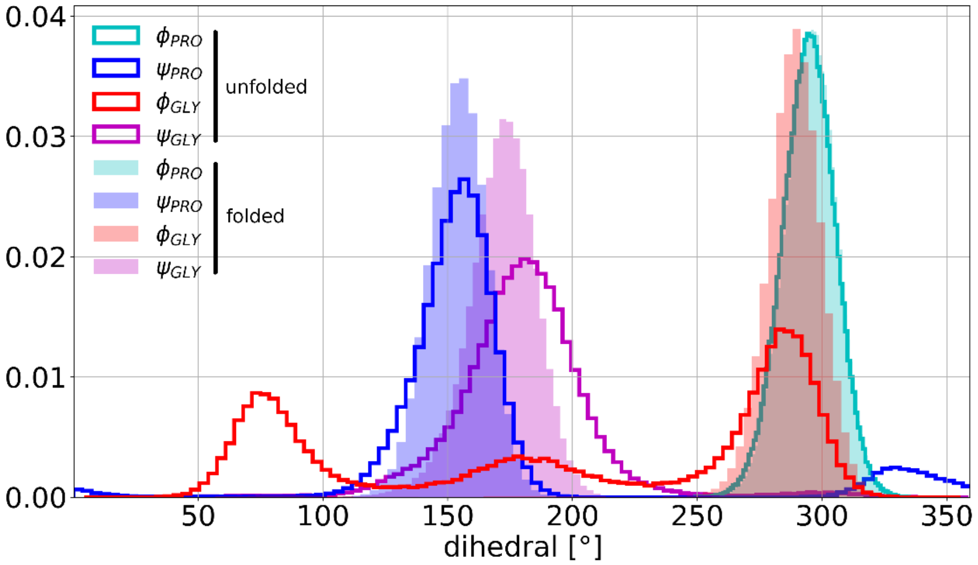

S7_Fig. Distribution of dihedral angles for two cases. In the folded case (filled areas) all angles populate a single state. In an unfolded case (framed areas) especially the ϕ-angle of GLY shows a broad variation. The ψ-angle of GLY is slightly shifted. The ϕ-angle of PRO does not change due to its ring structure, whereas the ψ-angle shows a second state around 330°.
