# Supplementary material for: Mechanism of collagen folding propagation studied by Molecular Dynamics simulations
Source: PLoS Comput Biol. 2021 Jun 8;17(6):e1009079. doi: 10.1371/journal.pcbi.1009079 (PMC8224937; doi:10.1371/journal.pcbi.1009079)
Supplement: S9 Fig — (PDF) [file pcbi.1009079.s009.pdf]

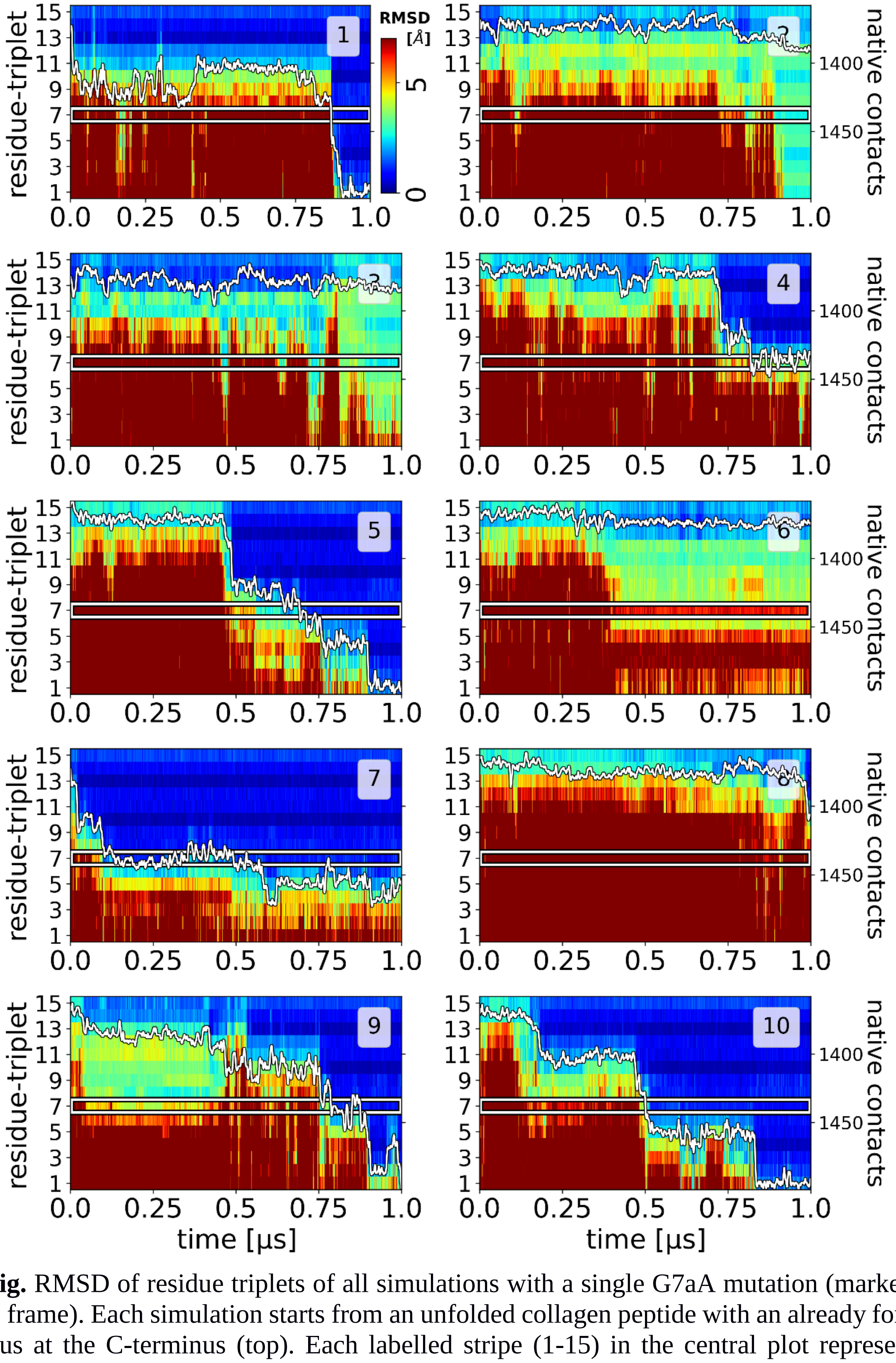

S9_Fig. RMSD of residue triplets of all simulations with a single G7aA mutation (marked by white frame). Each simulation starts from an unfolded collagen peptide with an already formed nucleus at the C-terminus (top). Each labelled stripe (1-15) in the central plot represents a residue triplet along the three strands. The RMSD of each residue triplet relative to the native folded structure is indicated by a color-code (color bar in the first plot). A blue color represents sampled states close to native, whereas red color corresponds to an unfolded triplet structure. The y-axis on the right side of the plot represents the number of native contacts between the three strands (white line in the plot).
