# Supplementary material for: Mechanism of collagen folding propagation studied by Molecular Dynamics simulations
Source: PLoS Comput Biol. 2021 Jun 8;17(6):e1009079. doi: 10.1371/journal.pcbi.1009079 (PMC8224937; doi:10.1371/journal.pcbi.1009079)
Supplement: S10 Fig — (PDF) [file pcbi.1009079.s010.pdf]

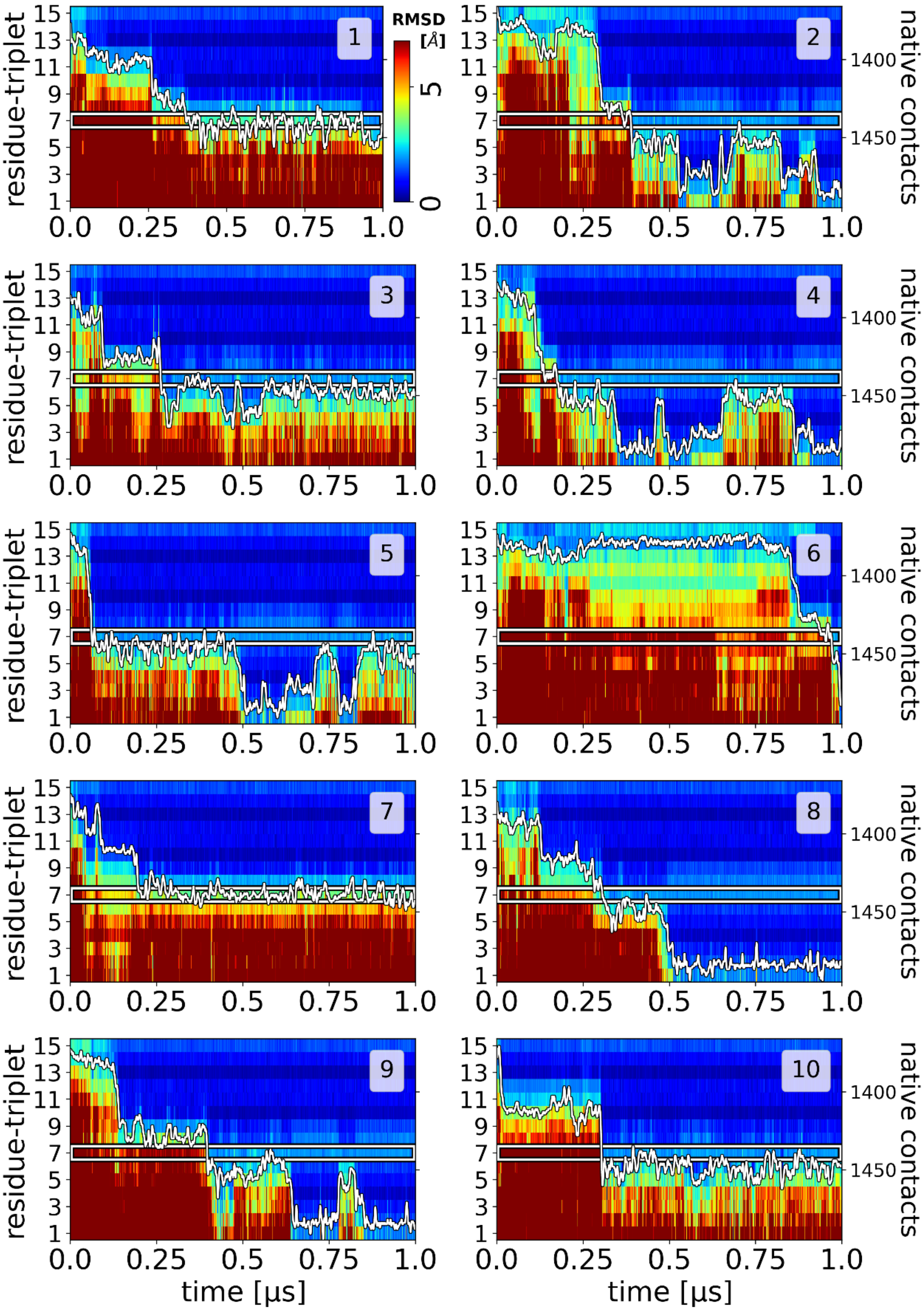

S10_Fig. RMSD of residue triplets of all simulations with a single G7aT mutation (marked by white frame). The simulation starts from an unfolded collagen peptide with an already formed nucleus at the C-terminus (top). See legend of S9_Fig.
