# Supplementary figures and images for: Mechanism of collagen folding propagation studied by Molecular Dynamics simulations
Source: PLoS Comput Biol. 2021 Jun 8;17(6):e1009079. doi: 10.1371/journal.pcbi.1009079 (PMC8224937; doi:10.1371/journal.pcbi.1009079)

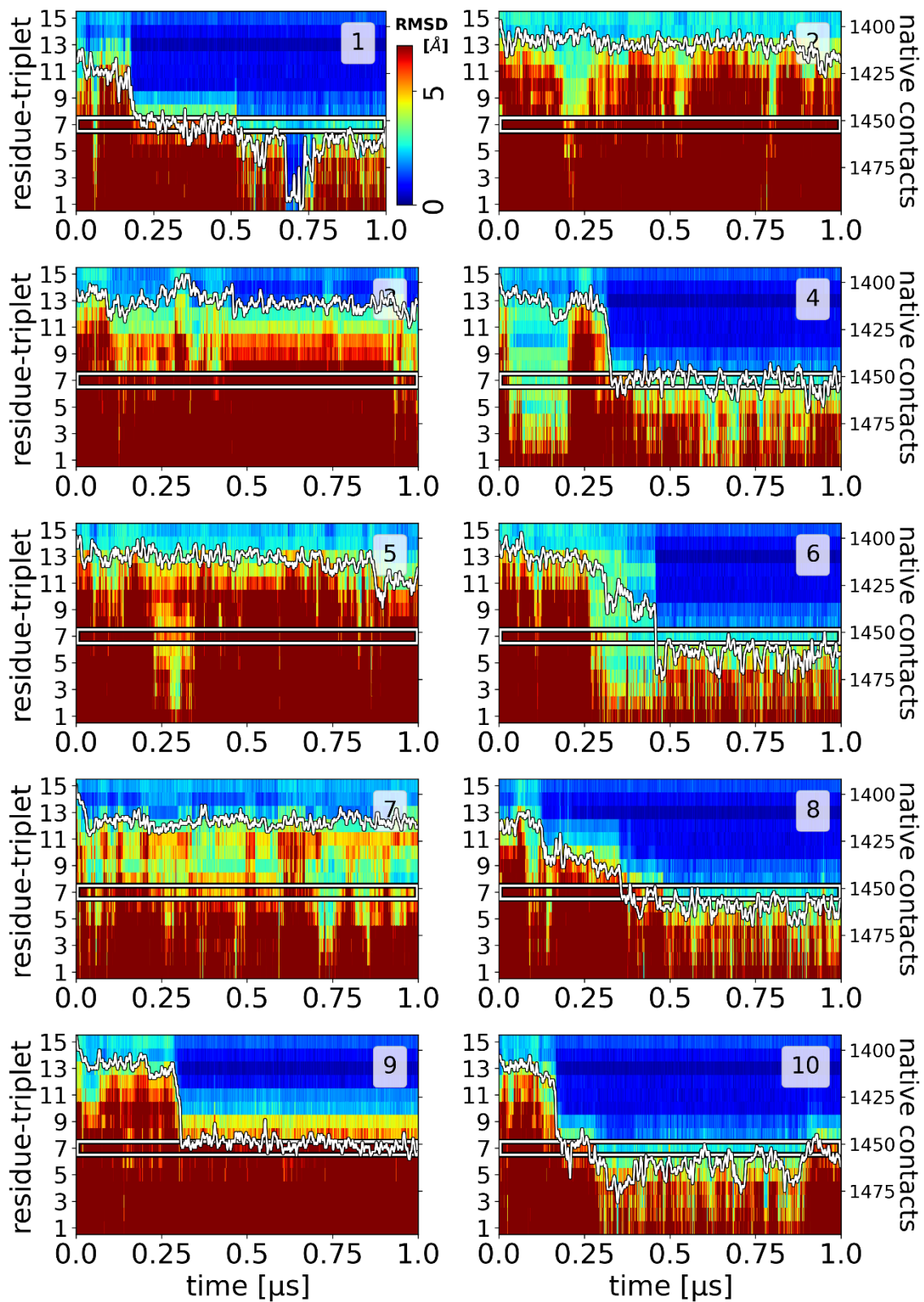

**S12\_Fig.** Same as S11\_Fig but for simulations with three mutations G7abcT (marked by white frame).

Supplement: S12 Fig — (PDF) [file pcbi.1009079.s012.pdf]
