# Supplementary material for: Mechanism of collagen folding propagation studied by Molecular Dynamics simulations
Source: PLoS Comput Biol. 2021 Jun 8;17(6):e1009079. doi: 10.1371/journal.pcbi.1009079 (PMC8224937; doi:10.1371/journal.pcbi.1009079)
Supplement: S13 Fig — (PDF) [file pcbi.1009079.s013.pdf]

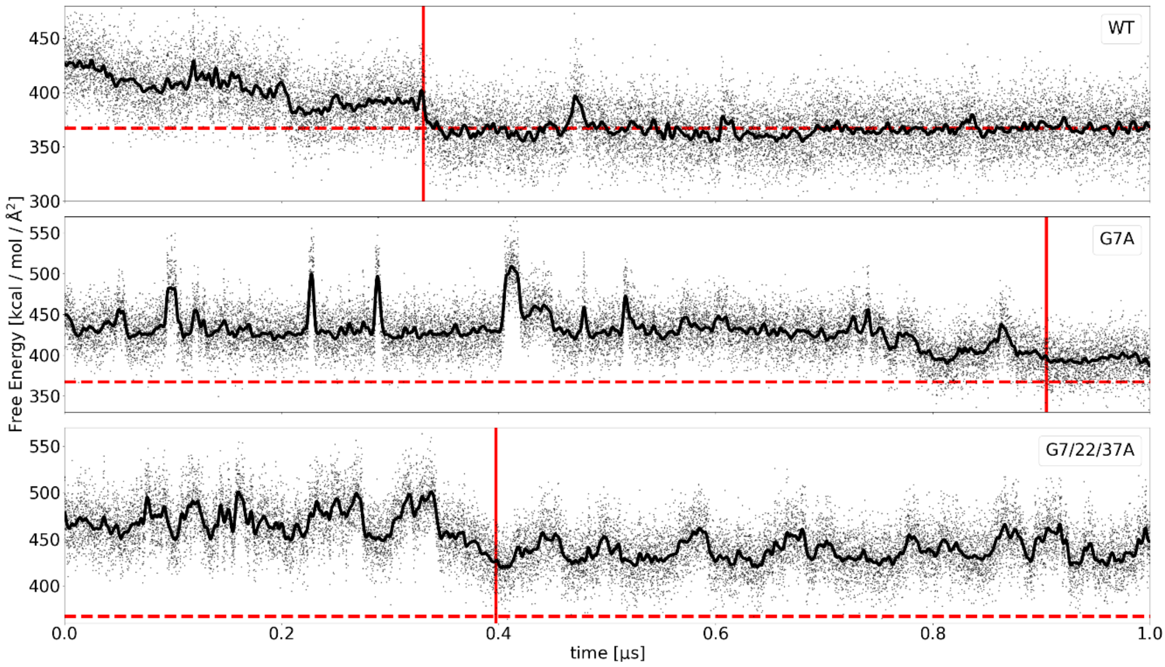

S13_Fig. Time course of the MMGBSA energy during three exemplary simulations. Red vertical lines indicate the time point when the RMSD of the N-Terminus reached values of a folded helix for the first time. The red dashed line displays the mean energy of a folded helix. In the first example a successful folding process of a wild-type peptide is shown (Figure 1 and S4_Fig (example 7)). The second example presents a peptide with one G7aA mutation in one strand (the letter a indicates chain A of the triple helix), which folded around 900ns, but the final energy was increased compared to a folded wild-type. The third example shows a peptide with three mutations (at position 7 of each chain) which temporarily got close to the folded state, but unfolded subsequently.
