# Supplementary material for: Mechanism of collagen folding propagation studied by Molecular Dynamics simulations
Source: PLoS Comput Biol. 2021 Jun 8;17(6):e1009079. doi: 10.1371/journal.pcbi.1009079 (PMC8224937; doi:10.1371/journal.pcbi.1009079)
Supplement: S1 Table — (PDF) [file pcbi.1009079.s014.pdf]

S1_Table. Free energy differences between beginning and end of simulations calculated using the MMGBSA method (kcal/mol)

Simulation	wildtype	wildtype/weak restraints	G7aA	G7aT	G7abcA	G7abcT
1	-70.7	-36.1	-80.2	-58.6	-57.8	-21.7
2	-82.3	-57.9	-76.5	-72.9	-57.4	-6.6
3	-94.0	-89.9	-36.2	-49.9	-16.6	-40.9
4	-68.9	-70.8	-56.8	-90.9	-58.6	-41.4
5	-104.8	-64.3	-73.9	-64.3	-44.4	-78.0
6	-87.4	-77.7	-28.4	-92.0	-51.9	-62.3
7	-77.9	-25.9	-66.3	-50.7	-44.5	-25.6
8	-68.3	-19.0	-58.7	-86.3	-68.6	-36.2
9	-81.3	-74.5	-95.8	-101.7	-38.6	-28.0
10	-92.0	-91.6	-71.1	-54.7	-62.6	-32.8
average	-83 ± 11	-61 ± 24	-64 ± 19	-72 ± 18	-50 ± 14	-30 ± 16
