# Supplementary material for: Mechanism of collagen folding propagation studied by Molecular Dynamics simulations
Source: PLoS Comput Biol. 2021 Jun 8;17(6):e1009079. doi: 10.1371/journal.pcbi.1009079 (PMC8224937; doi:10.1371/journal.pcbi.1009079)
Supplement: Attachment — Submitted filename: response_JH_MZ.pdf [file pcbi.1009079.s015.pdf]

Technische Universität München

T 38
Lehrstuhl für theoretische
Physik - Molekulardynamik

Technische Universität München · Lehrstuhl für theoretische Physik-
Molekulardynamik T38 · James-Franck-Str. 1 · 85748 Garching ·

PLOS Computational Biology
Editorial Board

Prof. Dr. Martin Zacharias

James-Franck-Str. 1
85748 Garching
Germany

Tel.: +49 (0) 89 28912335
Fax: +49 (0) 89 28912444

zacharias@tum.de
www.t38.ph.tum.de

Garching 8. 5. 2021

Dear Dr. MacKerell, dear Dr. Elofsson

Thank you for returning our manuscript entitled “Mechanism of collagen folding propagation studied by Molecular Dynamics simulations" by J. Hartmann and M. Zacharias and the comments of the reviewers. In the following we like to comment on the concerns of the reviewers and indicate the changes and additions we have made to the manuscript. We also provide a version of the manuscript with all changes marked red.

Reviewer #1: The paper by Hansmann and Zacharias is a very nice MD simulation effort to follow folding propagation from a preformed nucleus in atomic detail. They apply theirs study to collagen and observe the triple helix folding propagate involving first two chains forming a short transient template. Then three residues of the third chain fold on this template. They also observe the formation of loops with multiples of the repeating unit as a characteristic misfolding event.

1. The paper is very nice, competently carried out and well-written. Considering the importance of nucleus formation/disruption, in the discussion the authors could just briefly comment, as a future perspective, on the possible applicability to their trajectories of methods to find folding nuclei from simulations, such as the ones described in 10.1021/acs.jctc.0c00524 or 10.1021/acs.jcim.9b00588

<u>Response:</u> We thank the reviewer for the encouraging comment. We have added the perspective of detecting folding nuclei for collagen folding initiation using the recently developed technique mentioned by the reviewer. The changes are marked red in the revised version.

Reviewer #2: Understanding collagen folding and misfolding is of the utmost importance for human health, as it is associated to several diseases (especially vascular and skin diseases, but also arthritis, sclerosis and other very serious

auto-immune diseases for which we do not have cures yet, but only palliative drugs that reduce pain). Its understanding is still very limited and this paper is a definite step forward.

Publish as it is with highest recommendation in PLoS Computational Biology.

This is an excellent manuscript on the collagen folding investigated with state-of-the-art molecular dynamics. It provides an accurate view of collagen fibers misfolding, in agreement with kinetic experiments.

Response: We thank the reviewer for the encouraging comment.

1. As a minor: the authors could think changing the palette for the spectrograms, since the current palette disturbs the vision (especially in Figures 6 and 8).

Response: We have tried to modify the palette for the spectrograms shown in the Figures. However, we did not find a coloring that improved the vision. The current choice of more red-type "warm" towards "blue" type cold colors seem to be optimal. Other choices that we tried (including a more red-green contrast) did not improve the vision. Hence we decided to keep the current coloring scheme.

Reviewer #3: Collagen is a key component of the extracellular matrix and represents the most abundant protein of the human body. Collagen exhibits a characteristic fibrillar structure, in which three peptide chains form an elongated triple helix. There is a strong sequence preference for glycine at every third position, whereas the remaining positions are mainly occupied by proline or hydroxyproline. Due to this high sequence conservation, mutations in collagen are frequently associated with diseases. However, the despite its functional importance, the details of the collagen folding pathway are only poorly understood to date.

In their manuscript, Hartmann and Zacharias have used microsecond molecular dynamics simulations to study collagen folding. The simulations were performed at high technical standard (10 copies per simulation condition) and the use of harmonic restraints for the C-terminus has been carefully evaluated.

The simulations started from a folded C-terminal nucleus, with the remaining parts of the peptide chain unfolded. By monitoring the progression of triple helix formation, the authors were able to delineate a mechanism in two chains transiently first form a template to which the third chain attaches. This process occurs sequentially (units of three residues) on a timescale of approx. 75 ns per unit. Substitution of glycine by alanine or threonine decreased the stability and folding rates of the fibril and was also a major cause of fibril misfolding.

In summary, this is a comprehensive and technically sound study, which provides a wealth of novel structural information on the collagen folding process and the mechanism by which mutations cause collagen misfolding.

Response: We thank the reviewer for the encouraging comment (no changes are requested).

Finally, we like to thank the reviewers for the fair comments and hope that with the additions and changes we have made to the manuscript it is now acceptable for publication.

Yours sincerely,

Martin Zacharias
